# Nuclear and Mitochondrial Phylogenomics of the Sifakas Reveal Cryptic Variation in the Diademed Sifaka

**DOI:** 10.3390/genes13061026

**Published:** 2022-06-07

**Authors:** Melissa T. R. Hawkins, Carolyn A. Bailey, Allyshia M. Brown, Jen Tinsman, Ryan A. Hagenson, Ryan R. Culligan, Adena G. Barela, Jean C. Randriamanana, Jean F. Ranaivoarisoa, John R. Zaonarivelo, Edward E. Louis

**Affiliations:** 1 Department of Vertebrate Zoology, Division of Mammals, National Museum of Natural History, 10th and Constitution Ave, NW, Washington, DC 20560, USA; 2 Department of Conservation Genetics, Omaha’s Henry Doorly Zoo & Aquarium, 3701 S 10th St, Omaha, NE 68107, USA; carolyn.bailey@omahazoo.com (C.A.B.); ally.brown@omahazoo.com (A.M.B.); jen.tinsman@omahazoo.com (J.T.); ryan.hagenson@gmail.com (R.A.H.); rrculligan@gmail.com (R.R.C.); adena.barela@gmail.com (A.G.B.); kelynews1@yahoo.com (E.E.L.J.); 3 Madagascar Biodiversity Partnership, VO12 Bis a Manakambahiny, Antananarivo 101, Madagascar; genetics@omahazoo.com; 4 Anthropobiologie et Développement Durable, Faculty of Sciences, University of Antananarivo, Antananarivo 101, Madagascar; rjeanfreddy@gmail.com; 5 Faculty of Sciences, Président du Collège des Enseignants, University of Antsiranana, Antsiranana 201, Madagascar; zaonarivelo@yahoo.fr

**Keywords:** ecological niche modeling, genomics, Indriidae, Madagascar, *Propithecus*, Ultraconserved Elements

## Abstract

The most comprehensive phylogenomic reconstruction to date was generated on all nominal taxa within the lemur genus *Propithecus*. Over 200 wild-caught individuals were included in this study to evaluate the intra and interspecific relationships across this genus. Ultraconserved Elements (UCEs) resulted in well-supported phylogenomic trees. Complete mitochondrial genomes (CMGs) largely agreed with the UCEs, except where a mitochondrial introgression was detected between one clade of the diademed sifaka (*Propithecus diadema*) and the Milne-Edwards sifaka (*P. edwardsi*). Additionally, the crowned (*P. coronatus*) and Von der Decken’s (*P. deckeni*) sifakas belonged to a single admixed lineage from UCEs. Further sampling across these two species is warranted to determine if our sampling represents a hybrid zone. *P. diadema* recovered two well-supported clades, which were dated and estimated as being ancient as the split between the Perrier’s (*P. perrierii*) and silky (*P. candidus*) sifakas. The reconstructed demographic history of the two clades also varied over time. We then modeled the modern ecological niches of the two cryptic *P. diadema* clades and found that they were significantly diverged (*p* < 0.01). These ecological differences result in a very limited zone of geographic overlap for the *P. diadema* clades (<60 km^2^). Niche models also revealed that the Onive River acts as a potential barrier to dispersal between *P. diadema* and *P. edwardsi*. Further taxonomic work is required on *P. diadema* to determine if its taxonomic status should be revised. This first genomic evaluation of the genus resolved the relationships between the taxa and the recovered cryptic diversity within one species.

## 1. Introduction

Sifakas (genus *Propithecus*, family Indriidae) are a group of nine Critically Endangered diurnal lemur species distributed across Madagascar [1,2]. Four of these species transitioned from Endangered to Critically Endangered in 2020 (*P*. *coquereli*, *P. deckenii*, *P. coronatus*, and *P. verreauxi*), highlighting the dire conservation status of these primates. Several studies have sought to clarify the taxonomic relationships between all nine species; however, these were either based exclusively on mitochondrial sequence data, had small sample sizes, or did not include all nominal taxa [3,4]. This has left many questions unanswered across the genus.

Extensive inter- and intraspecific pelage variation [5,6,7,8,9,10], variable mating systems [11,12,13,14,15,16], and behavioral differences [16,17,18,19,20] have been characterized within the genus; however, studies are often limited to surveys spanning a short time period, single populations, or a combination of both. Some species also have sympatric ranges, and it appears that hybridization occurs in those regions [8,21,22]. These confounding factors have prevented many cohesive, wide-ranging evaluations of these taxa. Additionally, the debate around the number of species within the genus has not been resolved, and many publications rely on partial datasets where not all species/subspecies are represented or very limited molecular sequences are analyzed [23].

The biogeography of Madagascar is complex, and, as sifakas are distributed in most forested habitat types across the island, understanding its biogeographic patterns has broad implications for understanding the evolutionary history of forest-dwelling vertebrates across Madagascar. An east-–west divergence has long been identified with two major sifaka groups being recognized [3,4,24]. This biogeographic split was the basis for the original two species, *P. diadema* and *P. verreauxi*, when all other nominal taxa were listed as subspecies. Subsequently all subspecies were elevated to full species except *P. homeleas*, which was synonymized with *P. edwardsi* [3,25]. Additionally, *P. tattersalli* was only described to science in 1988. This species is morphologically similar to the western species despite its distribution in northern Madagascar. Currently, the western groups include *P. verreauxi*, *P. coronatus*, *P. deckenii*, *P. coquereli*, and, more distantly, *P. tattersalli*, while the eastern groups include *P. diadema*, *P. perrieri*, *P. candidus*, and *P. edwardsi*.

Beyond an east–west gradient, Madagascar has many more restricted biogeographic regions [26]. A broad-scale evaluation of many populations of sifakas across Madagascar will allow a comprehensive assessment of biogeographic hypotheses and subsequent impacts on species boundaries and speciation dynamics. Two major hypotheses exist, the current climate hypothesis [26] and retreat-dispersion watersheds hypothesis [27]. The current climate hypothesis splits Madagascar into 14 climate zones, where some regions of the island represent consistent, broad climate zones, e.g., southwest Madagascar contains only four zones, and others (particularly the eastern coast of Madagascar) contain minimally six long narrow climate zones spanning the contrasting elevational and ecological gradients along eastern Madagascar.

While debates continue about whether the number of primate species is inflated or is representative of cryptic diversity [23,28], habitats are being degraded, the climate is changing, and populations are shrinking across the island [29,30,31,32]. Some *Propithecus* populations have been hunted to extirpation and required translocations to reestablish populations in newly protected areas [33]. One study evaluated the demographic changes from the Holocene–present within two sifakas and recovered that historical and modern pressures both likely had an effect on the reduction in population sizes [34]. Additionally, [35] used whole genome sequences of four species to look at deeper demographic histories and found variable patterns of expansion and contraction across the island, but the study was lacking several species and had limited sample sites. Previous studies into the genus revealed that several species have suffered from population bottlenecks based on microsatellite and genomic analyses [34,36,37] and also identified several important reservoirs of non-bottleneck populations, which have subsequently become extirpated due to a lack of habitat protection (e.g., Bora forest population of *P. coquereli*, [36]). As such, it is imperative to understand where the remaining strongholds of genetic diversity are found to ensure the long-term survival and perpetuation of these endangered species.

In this paper, we incorporate large, island-wide sampling and include all species of *Propithecus* from as many populations as possible including over 200 wild-caught individuals, to fully reconcile the phylogenomic relationships of this genus. This comprehensive phylogenomic reconstruction can be used to identify genetic divergence at the inter- and intraspecific levels. To accomplish this, we enrich thousands of nuclear markers (Ultraconserved Elements, UCEs hereafter; [38]) and sequence complete mitochondrial genomes (CMGs) to provide the first nuclear phylogeny of this group and the largest mitochondrial dataset to date. We also used these data to date the split between and within species and provide insights into the biogeographical processes that shaped this genus.

## 2. Materials and Methods

### 2.1. Samples

A total of 208 wild, free-range sifakas were sampled for this study, spanning all nominal species with a broad geographic spread. Details of in-group individuals and out-groups are summarized in Table 1 and expanded in Supplementary Table S1. All individuals were collected from field expeditions sampled between the years 1999–2008. Lemurs were immobilized with a CO_2_ Dan-Inject projection rifle (Knoxville, TN, USA) using Pneu-darts™ (Williamsport, PA, USA) with 10 mg/kg of Telazol (Zoetis, Parsippany, NJ, USA) for the estimated weight of each individual. Following immobilization, blood and tissue samples (four 2 mm diameter ear pinnae biopsy punches) were collected and stored in appropriate storage buffers and kept at −20 °C while in the country and at −80 °C following exportation [39]. The samples were ultimately exported to the genetics laboratory at Omaha’s Henry Doorly Zoo and Aquarium (Omaha, NE, USA) in compliance with animal care and use, collection, and export permits. While sedated, the animals had a HomeAgain microchip (Merck & Co., Inc., Kenilworth, NJ, USA) placed subcutaneously between the scapulae to enable identification if individuals were recaptured. GPS locations of each individual were recorded in the field, and the animals were released at their collection locations following their recovery from the sedative. All handling procedures followed the guidelines of the American Society of Mammologists [40].

### 2.2. Data Generation and Sequencing

Following the selection of individuals, tissue samples were extracted using phenol-chloroform isoamyl isolation following [41], as performed in [42]. Approximately ¼ of a single ear punch was digested for each individual, with the goal of obtaining 500 ng of starting material. Following DNA isolation, samples were sheared to approximately 300 bp with a Covaris M220 (Woburn, MA, USA) using the default shearing protocol. Following sonication, a 1X Solid Phase Reversible Immobilization (SPRI hereafter) cleanup was performed on each sample using the protocols detailed in [43]. Cleaned and sonicated products were visualized on a Fragment Analyzer using the High Sensitivity NGS kit (Agilent Technologies Inc., Santa Clara, CA, USA). Dual indexed Truseq-style indices were ordered from the UGA sequencing core [44], and a library preparation was performed with a KAPA library preparation kit (Roche, Basel, Switzerland). A complete list of barcode combinations for each sample is provided in Supplementary Table S2. Modifications to the manufacturer’s protocol included ¼-sized reactions as detailed in [45]. Amplification with the i5 and i7 adapters was performed for 10 cycles following the KAPA Biosystems protocol in 50 µL reactions using KAPA High Fidelity Taq in reactions containing 25 µL of KAPA HiFi 2X ReadyMix Taq, 15 µL of adapter ligated DNA, 7.5 µL of ddH20, and 1.25 µL of each i5 and i7 adapter (which add the unique barcodes and have the PCR priming site) under the following cycling conditions 98 °C for 45 s for an initial denaturation step, followed by ten cycles of 98 °C for 15 s, 60 °C for 30 s, 72 °C for 60 s, with a final extension of 72 °C for 5 min. Following amplification, a 1X SPRI bead cleaning was performed, and the purified products were eluted in distilled water. The purified products were quantified with a Qubit 2.0 fluorometer using the high sensitivity kit (ThermoFisher Scientific, Waltham, MA, USA).

In-solution enrichments were performed on each sample for a set of 5k tetrapod UCEs, and whole mitochondrial genomes were mined from bycatch sequences recovered from the raw enriched sequences [38,46]. The Ultraconserved Elements are well characterized markers distributed across the genome, yet specific details about the functionality of the UCEs are less established [46]. Most studies suggest UCEs have a functional role in regulating splicing and epigenetics [47]. In general, UCEs are short (under 200 bp), ultra-conservative fragments of the genome, which, as such, are used for anchoring probes across diverse taxa. Phylogenetic informative sites are recovered from the variable flanking regions, which increase in variation with distance from the core UCE. One major utility is that a species-specific marker design is not necessary, and they have proven useful in various evolutionary scales spanning phylogenomics to population genomics [45,48].

Additionally, a custom gene set was designed based on a myriad of genes for phylogenetic reconstruction. However, only three genes were mined from this dataset for specific analyses, which is detailed below. RNA probes were synthesized by MYbaits (Daicel Arbor Biosciences, Ann Arbor, MI, USA), and the enrichment conditions followed manufacturer’s protocols from version 3.01 with the following modifications: hybridizations occurred at 60 °C and washed with Wash Buffer 2 (as opposed to Wash Buffer 2.2). All enrichments contained multiplexes of 3–4 samples per enrichment, totaling 500 ng of input DNA (125 or 166 ng of DNA for each sample).

Following enrichment, pools were amplified for 12 cycles using Illumina primers (i5: 5′-AATGATACGGCGACCACCGAGA*T-3′ and i7: 5′-CAAGCAGAAGACGGCATACGAGA*T-3′) with a five-minute denaturation at 95 °C and a thirty-second anneal at 60° C followed by a five-minute final extension at 72 °C. After amplification, another 1X SPRI purification was completed, and the captured products were visualized on the Fragment Analyzer, again using the high-sensitivity NGS kit.

A quantitative PCR was completed on an ABI Quantstudio 3 (ThermoFisher) using KAPA Illumina library quantification kit (SYBR green fluorescence), using 10 µL reactions with ROX as a passive background dye. Enrichment pools were then combined in equimolar ratios in preparation for sequencing. Sequencing was performed via the Discovery Life Sciences sequencing core (Huntsville, AL, USA) on a single run of an Illumina NextSeq 550 (Illumina, San Diego, CA, USA) using 2 × 150 bp PE sequencing.

Three additional species were included as out-groups, *Lemur catta*, *Indri indri*, and *Avahi peyrierasi*, to allow complete inference to genera within the Family Indriidae. We mined raw (unpublished) genomic data for the UCEs for these taxa.

### 2.3. Data Processing: UCEs and CMGs

The UCE results were analyzed following the phyluce pipeline v1.5.0, available at: http://phyluce.readthedocs.io/en/latest/ (accessed on 1 January 2020) [49]. Quality control was performed with Trimmomatic v0.36 [50], and Trinity v2.5 [51] was used for de novo assembly following the phyluce documentation.

For phylogenetic reconstructions, we decided to first test trees generated with the fifty ‘most informative’ UCE loci as determined by a script from the phyluce pipeline (get_informative_sites.py), which details the length, number of variable sites, and number of phylogenetically informative sites across all enriched UCEs. Additional subdivisions of the dataset were performed to test for differences based on the loci included.

Complete mitochondrial genomes (CMGs hereafter) were extracted from the UCE sequence data from sifakas (byproduct sequences from each enrichment) and mapped to a published reference sequence from a closely-related species. Eastern sifakas (*P. diadema*, *P. candidus*, *P. perrieri*, and *P. edwardsi* were mapped to a diademed sifaka from Zahamena National Park (GenBank Accession number: KJ944257). Western species (*Propithecus tattersalli*, *P. verreauxi*, *P. coronatus*, *P. deckenii*, and *P. coquereli* were mapped to a published Coquerel’s sifaka (GenBank Accession number: AB286049). Read mapping was performed using bwa v0.7.10 via the ‘mem’ command, all BAM files were imported to Geneious v10.2.6 [52], and only mitogenomes with greater than 5× average coverage were used in subsequent analyses (details can be found in Supplementary Table S3). Consensus sequences were extracted, and all mitogenomes were aligned with MAFFT v7.222 [53] using the ‘L-ins-I’ algorithm through the Geneious plugin. Comparisons with published sequences of mitochondrial genes (specifically cytochrome *b*) were performed on gene extractions from the completed mitogenome datasets to allow for fine-scale intraspecific inferences and to avoid issues with branch lengths when comparing incomplete datasets.

### 2.4. Data Partitioning and Phylogenomic Inference

Ultraconserved Elements have unique properties different from many other nuclear markers. The in-solution enrichments bind target molecules by a probe designed around a conserved portion of the genome (in our case, conserved across tetrapods). The flanking sequences of these markers contain the phylogenetically informative sites, and, as such, have a much different rate of molecular evolution from the conserved portion. A number of phylogenies incorporating UCEs have tested variable partitioning with debatable success [38,45,46,48,54]. Based on these results, novel methods of data partitioning were devised particularly for UCE loci [55]. Here, we follow Tagliacollo and Lanfear [55], incorporating the SWSC-entropy model of partitioning by evaluating the AIC values for the more variable flanking sequence from the core.

Due to specific compilation issues, the script was modified slightly and incorporated the best partitioning scheme identified in [55] for our dataset [56]. Following SWSC-entropy modeling, the partitions were run through PartitionFinder v2.0 [57] to determine the best substitution models. We ran our analysis on linked branch lengths and AICc models, only incorporating models from MrBayes v3.2.6 [58]. We also selected the ‘rcluster’ model and used the ‘–raxml’ flag during the run to allow for faster completion.

Gene trees were generated for all loci with more than 100 individuals (4176 loci), exact sequence matches were removed with a Sequence Dereplicator and Database Curator (SDDC) v1.0 (available at: https://github.com/Eslam-Samir-Ragab/Sequence-database-curator) (accessed on 1 January 2020) using the ‘derep’ option. Each locus was run through RAxML v8.2.7 [59] (through the Geneious Plugin), with a GTR γ nucleotide model, with rapid bootstrapping and searching for the best-scoring ML tree, with 100 bootstrap replicates.

Two subsets of UCE loci were tested for topological congruence to allow for the use of more complex programs on reduced datasets for particular analyses. Subsets included the 50 ‘most informative loci’ (MI hereafter) per the number of informative and variable sites from the phyluce scripts, as well as by the number of loci with 95% of all represented taxa. In order to evaluate the performance of various data partitions, the amount of missing data was also considered, as missing data can influence the branch length and topology during phylogenetic reconstruction.

In addition to the SWSC-entropy modeling, we ran the 50 MI UCE dataset through IQ-TREE [60] using the W-IQ-TREE server [61]; this uses 1000 ultrafast bootstrap replicates and the Bayesian Information Criterion (BIC) following [62] to test for model selection.

The 95% complete dataset was concatenated and run through various phylogenetic tree software to evaluate topological congruence with other data partitions. This dataset was edited to remove large stretches of missing data and stretches of identical sequences as that could significantly affect the resulting topology and branch lengths, and the starting length added a computational complexity, preventing analysis on most computers. After cleaning the data, the resulting alignment was 60,308 bp long. A UPGMA tree was run using the Geneious Tree Builder tool using Jukes–Cantor distance models with 10 different bootstrap replicates. Additionally, the same dataset, but with a reduced number of individuals, was run to evaluate interspecific patterns across a dataset with fewer individuals (randomly selected individuals or individuals with large proportions of missing data were removed) to allow for Bayesian Inference. The MrBayes run (via the Geneious plugin) had 1,100,000 generations, with a subsampling frequency every 2000 chains. A GTR substitution model with γ rate variation was selected based on the PartitionFinder results (Supplementary Table S4). A total of 4 chains were run with a temperature of 0.2, and 100,000 generations were removed as burn-in on the reduced dataset of 79 individuals.

### 2.5. Species Tree Generation and Divergence Dating

NJst [63] and STAR were used to estimate the species tree from the 50 most informative UCE loci. They were performed using the STRAW webserver [64]. No data partitioning was completed on the alignments, and the RAxML trees from the dataset, including 95% of individuals (containing 512 UCE loci), were used for the input trees. All trees were rooted on the *L. catta* samples.

The program BEASTv1.8.4 [65] was used to date the divergence between species for a separate subset of individuals. A single representative of each species was included for all except for *P. diadema*, where two individuals were included to span the intraspecific variation observed in our initial phylogenetic analyses. A single out-group sequence was included, RANOL6, *A.* *peyrierasi*, to root the tree and add a calibration point based on three previous estimates [66,67,68]. From Herrera and Davalos [67], the root height of the *Avahi–Propithecus* split was estimated as having occurred 20.64–19.34 million years ago, as such the average of 19.99 MYA was used as a prior calibration point using a normal distribution with a 5% at 18.26 MYA and a 95% at 21.54 MYA. A separate analysis was performed using the splits estimated in Kistler et al. [68], as there are significant age discrepancies between the two datasets. The *Avahi–Propithecus* split for this dataset was estimated at 13.4 MYA. A third age estimate was incorporated from the 10kTrees v3 primate tree (available at: https://10ktrees.nunn-lab.org/Primates/ Downloaded 4 March 2021 for all Lemuriformes [66], which recovered a 16.5 MYA split between *Avahi* and *Propithecus*.

For all three different prior calibration datasets, an uncorrelated relaxed clock was used in addition to a Yule speciation process. The MCMC was run for 25 million generations, and data were logged every 1000 states. Three replicates of the BEAST analysis were run to assess convergence using the CIPRES Server [69], and TreeAnnotater v1.8.4 [65] was used to summarize the resulting trees where nodes with less than 0.9 posterior probability were discarded. Figtree v1.4.2 [70] was used to visualize the resulting tree.

### 2.6. Bayesian Skyline Plot Reconstructions

Intraspecific variation was modeled for taxa with deep divergence to evaluate each clade independently for evidence of variable coalescent histories using an Extended Bayesian Skyline Plot, EBSP hereafter, using BEAST v.2.6.6 [71]. A subset of loci from the 50 Most Informative UCEs was used for individuals from each clade. The eight loci included: four UCEs, 2642, 4557, 7310, and 7428; three nuclear genes (ABCA1, ADORA3, and RAG1); and the mitochondrial cytochrome *b* gene were included in this analysis for a total alignment length of 6511 bp. The UCEs were selected based on the number of representations of individuals from both clades 4 and 5 (to avoid issues associated with missing data), as well as completeness from the nuclear genes. Multiple types of genes were included to represent nuclear (UCEs and coding genes) as well as mitochondrial cytochrome *b* to avoid biases with a single type of marker. The same loci were used for all clades. A HKY substitution model was used for each gene. Initially, 10 million chains were run, with trees logged every 1000 chains. TRACER v1.7.1 [72] was used to visualize the log files and ensure adequate ESS; if the ESS was under 200, an additional run was performed with an increased number of chains. The R software v3.5.3 [73] was used to plot the EBSP log files following commands in the Extended Bayesian Skyline Plot tutorial (available online: http://evomicsorg.wpengine.netdna-cdn.com/wp-content/uploads/2015/11/ebsp2-tut1.pdf accessed on 1 January 2020). Plots were visualized using the defined functions in the R script file, “plotEBSP.R” from the tutorial. The four plots (see Supplementary Figure S1) included the Central Posterior Density (CPD), as opposed to the highest posterior density (HPD) intervals and traces for each replicate in the analysis.

### 2.7. Ecological Differentiation

*P.diadema* and *P. edwardsi* occur in lowland and medium-elevation moist evergreen forests stretching along much of eastern Madagascar [1,25]. To explore differences in ecological niches between intraspecific subdivisions, ecological niche models (ENM) were constructed and compared for clades within *P. diadema* and *P. edwardsi*. Additionally, potential contact zones were identified, and their geographical areas quantified by binarizing the models using Minimum Training Presence, a metric that yields the largest potential area of overlap. The eastern rainforest ecoregion was selected as the study extent [74].

We checked 19 climate variables from WorldClim 2.1 (resolution 30 arc seconds, ~1 km^2^; [75]) for correlation and retained variables with a correlation coefficient of Pearson’s |r| < 0.7 for subsequent analyses. The variables we used in our analyses were isothermality (BIO3), temperature seasonality (BIO4), maximum temperature of the warmest month (BIO5), precipitation of the wettest month (BIO13), and precipitation of the driest month (BIO14).

The occurrence records for the focal lineages were drawn solely from field expeditions conducted under the guidance of EEL. In addition to the *P. diadema* and *P. edwardsi* included in the genetic analysis (Table 1), other *Propithecus* individuals were immobilized between 1999–2015, and all associated GPS data were included for niche model estimations (Supplementary Table S1). We accounted for sampling bias in our occurrence records by drawing background points from a bias file representing our sampling effort across Madagascar. We used more [76] than 5000 lemur occurrence records, which were collected during field expeditions across Madagascar and a weighted Gaussian distance kernel with Brown and Yoder’s [77] maximum radial search distance as our bandwidth to create the sampling effort/bias file [78]. We drew 10,000 weighted background points from this sampling effort surface for niche model evaluation and construction.

We chose model parameters using ENMeval v2.0.3, an R package for evaluating goodness-of-fit and overfit in niche models [79]. We used the Maxent v3.4.3 [80] algorithm to build models [80]. All tests were conducted in R v4.0.5 [81]. We compared models with linear, quadratic, hinge, and/or product features, and regularization multipliers from 1–5, using block spatial partitions to partition test and training data for k-fold cross validation [79]. We were concerned about model overfitting due to our small sample size. Thus, to select model parameters, we prioritized the models with the best tenth percentile omission rates, a metric of overfit (best being the value closest to the expected 0.1) and from those chose the models with the highest average AUC [82,83]. Parameters for our best models can be found in Supplementary Table S3.

We used ENMTools v1.0.6 [84,85] to conduct niche identity tests, which evaluate the hypothesis that two species with the same extent of occurrence occupy the same ecological niche [84,85]. We compared the two *P. diadema* clades to each other, and we also compared both of these clades to *P. edwardsi* as an out-group. We used the model parameters we identified with ENMeval, defaulting to the simplest parameters when the two species being compared had different parameters (e.g., the fewest feature classes and the highest regularization multiplier). We compared empirical metrics of niche overlap for pairs of sifaka to niche overlap values for 99 ENMTools-generated pseudo-replicates for a *p*-value resolution of 0.01 [84,85].

We also ran an age–niche overlap correlation test for the genus *Propithecus*, which asks whether the amount of overlap between two species’ niches is associated with their phylogenetic relatedness using the dates from the 10kTrees phylogeny [66]. For that test, we used either the western and spiny ecoregions or the eastern ecoregion as the background from which to draw sampling-effort-corrected points depending on the species’ range, and we used the first six principal components of all 19 bioclimatic variables as our environmental variables.

## 3. Results

### 3.1. Ultraconserved Elements

The NextSeq run generated a total of 493,434,654 paired reads, with 458,618,594 successfully demultiplexed to a known barcode combination. Four individuals had poor sequence recovery (less than 40,000 reads), and three individuals, which appeared to have chimeric reads, were removed from subsequent analysis, resulting in 205 individuals for UCE analysis with an average of 2,226,106 PE reads per individual, detailed in Supplementary Table S4 (including the four ‘failed’ enrichments). The seven removed individuals represented replicated survey sites and were not replaced in this study. Following targeted enrichment of the in-groups and the shotgun sequenced out-groups, we recovered a total of 4583 UCE loci in the incomplete matrix. The total length of the incomplete matrix was 3,113,282 bp. The phyluce script ‘get informative sites’ found that, of the 4583 loci, only 48 loci did not contain any informative sites. When the minimum number of individuals per alignment was increased to 100, 4176 loci (with a total length of 2,953,642 bp) contained informative sites. Most loci had between 21–30 informative sites per alignment but ranged from 1–192 informative sites. These data are presented in Supplementary Figure S2.

**Figure 1:**
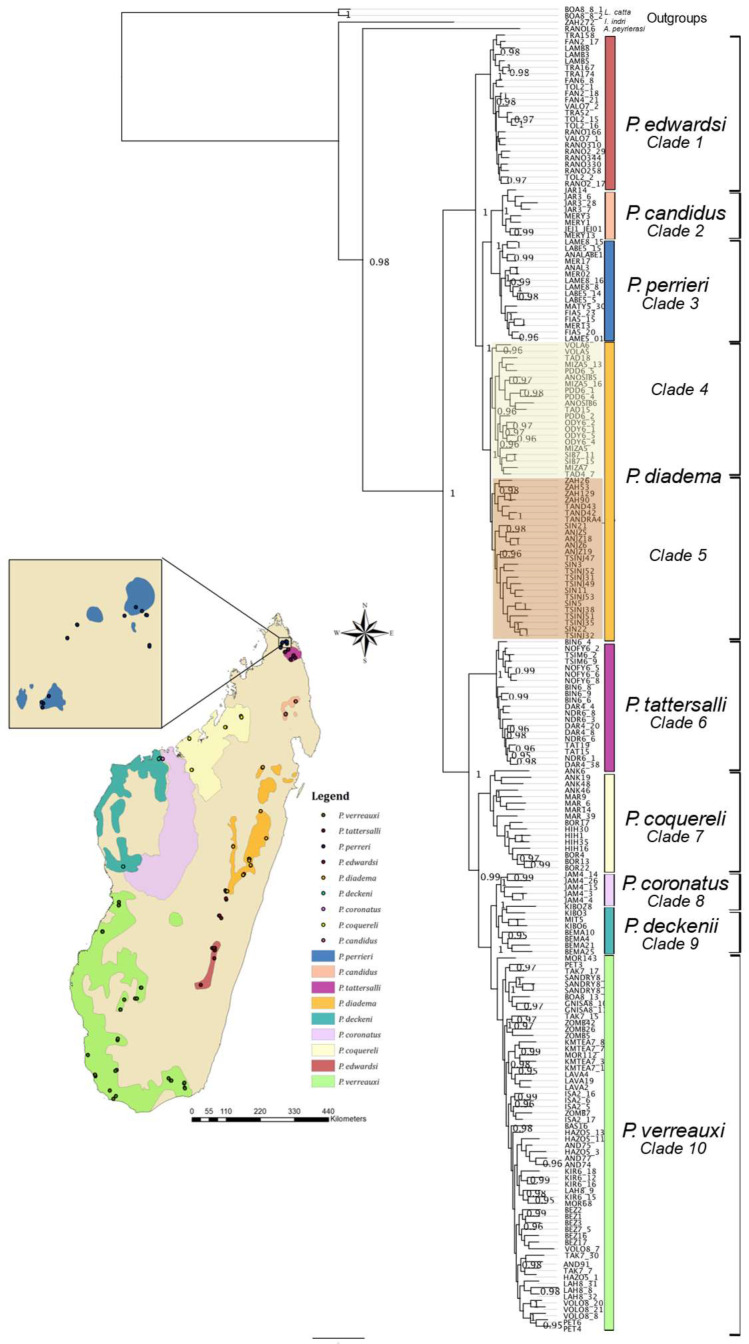
Distribution of all nine species of sifaka across Madagascar (range estimates obtained from IUCN, www.iucnredlist.org, accessed on 1 January 2020), with each individual included plotted in the matching color. Note the two clades within *P. diadema* are shown in light and darker orange shades. Phylogenetic tree generated in MrBayes from the 50 most informative UCEs. For simplicity, the major clades have been labelled as numbers 1–10 and colored the same across all figures.

The 50 MI UCE dataset included 205 individuals and had a total length of 39,966 bp. The 95% complete dataset included 193 individuals and 512 loci and had a total length of 425,485 bp when concatenated. Phylogenetic trees were computed from multiple data partitions, and only missing data appeared to strongly influence the tree topology. The 50 MI and the 95% complete datasets resulted in highly similar topologies, and, as such, the subset containing 50 loci was used for subsequent analysis, as it was computationally less intensive and provided the same results as larger datasets (Figure 1).

### 3.2. Data Partitioning and Phylogenetic Inference

Following the recovery of the 50 most informative UCE loci, we ran those loci through SWSC and PartitionFinder v2.0 and recovered 104 data partitions and nearly all recovered GTR + I + G as the best models. Only five of the 104 partitions selected the slightly different GTR + G as the best model (details provided in Supplementary Table S5).

Three data partitions were used for phylogenetic reconstructions, and each is described here. (1) A concatenated tree of the 50 most informative loci was compiled, which resulted in 39,966 bp in length, (2) a second containing 95% of all individuals present per locus, which resulted in 193 of the 203 samples 425,485 bp in length, and (3) one version of the 95% dataset ‘stripped’ of long stretches of missing and uninformative sites which was 60,308 bp long. In order to ensure the topology recovered from the 50 most informative UCE loci was consistent with other subsets of UCE loci, we ran species trees and concatenated trees from 512 loci to evaluate the similarity in topology. Additionally, a UPGMA tree was constructed using the Geneious tree builder [52] on data partition 2 (the 512 UCE dataset, 193 individuals, and 425,485 bp long alignment).

Topologies confirmed previous studies including [67] with *Indri* branching first, followed by *Avahi*, and *Propithecus* was monophyletic (trees were rooted on *L. catta*; Figure 1). Within *Propithecus*, the divergent east–west split was confirmed (posterior probability = 1.0), and the species composition was as predicted with *P. edwardsi* (pp = 1.0, clade numbers have been added to all figures and are consistently referenced hereafter, clade 1), *P. candidus* (pp = 1.0, clade 2), *P. perrieri* (pp = 1.0, clade 3), and *P. diadema* (clade 4 and 5, pp 1.0 for both) forming the eastern group, and *P. tattersalli* (pp = 1.0, clade 6), *P. coquereli* (pp = 1.0, clade 7), *P. coronatus* (pp = 1.0, clade 8), *P. deckenii* (pp = 1.0, clade 9), and *P. verreauxi* (pp = 1.0, clade 10) forming the western group. In the eastern clade, *P. diadema* formed two well-supported clades (clades 4 and 5), which prompted further evaluation.

### 3.3. Species Trees and Divergence Dating

Following species tree generation on the 512 loci (95% complete dataset), all previously designated species were supported, with the exception of *P. deckenii* and *P. coronatus*, where admixture was observed, which was expected based on field observations and intermediate appearing individuals. The species tree also recovered two clades within *P. diadema*; however, some populations were split among the two clades, which could be due to missing data or incomplete lineage sorting (Supplementary Figure S3).

**Figure 2:**
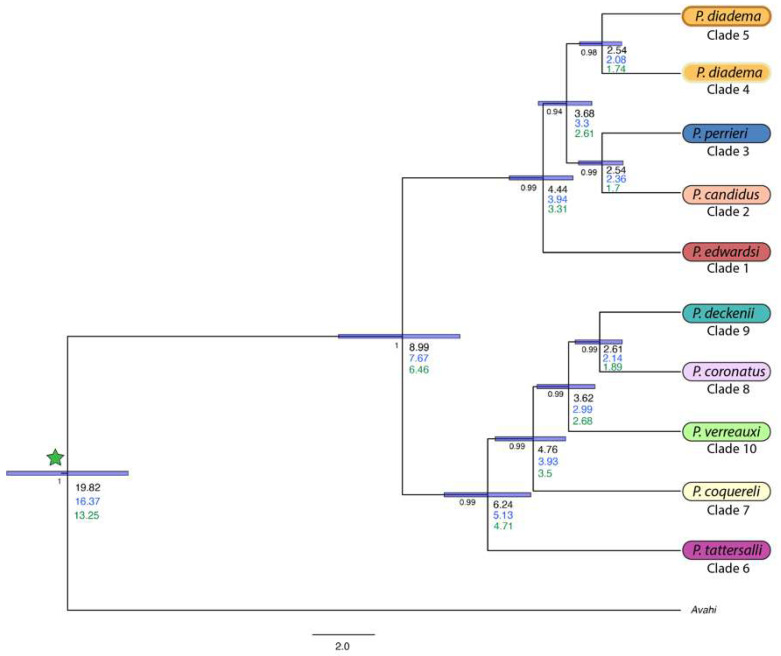
Divergence dated tree including UCE data generated in BEAST v1.8.4 [72]. Note the star at the root of the tree represents a calibration point as recovered from either [67]; the 10K primate tree [66] or [68], with different age estimates represented in black, blue, and green, respectively. Posterior probabilities are shown to the left of the ages (in millions of years) for all nodes. HPD bars shown were recovered from the [67] calibrated BEAST analysis.

BEAST v1.8.4 [65] was used to date the divergence of the UCE tree for the 50 MI loci at the species level and, as such, included 11 individuals. From the analysis incorporating a calibration point from [67], the following estimates were recovered. The crown of the genus *Propithecus* was dated to 19.82 MYA from the *Avahi* out-group (Figure 2). Within *Propithecus*, the well-established east–west group split was recovered at approximately 8.99 MYA and separated the western species *P. coquereli* (clade 7), *P. verreauxi* (clade 10), *P. coronatus* (clade 8), *P. deckenii* (clade 9), and *P. tattersalli* (clade 6), from the eastern species, *P. diadema* (clades 4 and 5), *P. perrieri* (clade 3), *P. candidus* (clade 2), and *P. edwardsi* (clade 1). Within the western group, *P. tattersalli* was the first species to diverge at 6.24 MYA, followed by *P. coquereli* at 4.76 MYA. *Propithecus verreauxi* branched next at 3.62 MYA. *Propithecus coronatus* and *P. deckenii* split at 2.61 MYA.

In the eastern group, the first species to diverge was *P. edwardsi* at 4.44 MYA, followed by the *P. diadema* split from *P. candidus* and *P. perrieri* at 3.68 MYA. *Propithecus candidus* split from *P. perrieri* around 2.54 MYA. Finally, we dated the split between the two recovered clades of the *P. diadema* at 2.54 MYA, equally as long ago as the split between *P. candidus* and *P. perrieri*. This is a significant finding and may have implications for the already Critically Endangered *P. diadema*.

Finally, the 10kTree age estimate puts the *Avahi–Propithecus* divergence at 16.5 MYA. The BEAST run recovered the root at 16.37 MYA, with an east–west divergence at 7.67 MYA, the dates recovered for each clade were about 10–20% younger based on this calibration point.

The replicates incorporating the dates obtained from Kistler et al. [68] recovered the following divergence date estimates. The *Avahi–Propithecus* split was estimated at 13.25 MYA, with the east–west split at 6.46 MYA. Across the tree, the splits ranged from 24–33% younger than Herrera and Davalos [67]. All dates recovered from the three calibration points [66,67,68] are presented in Figure 2, and a summary is presented in Table 2.

### 3.4. Mitochondrial Genomes

**Figure 3:**
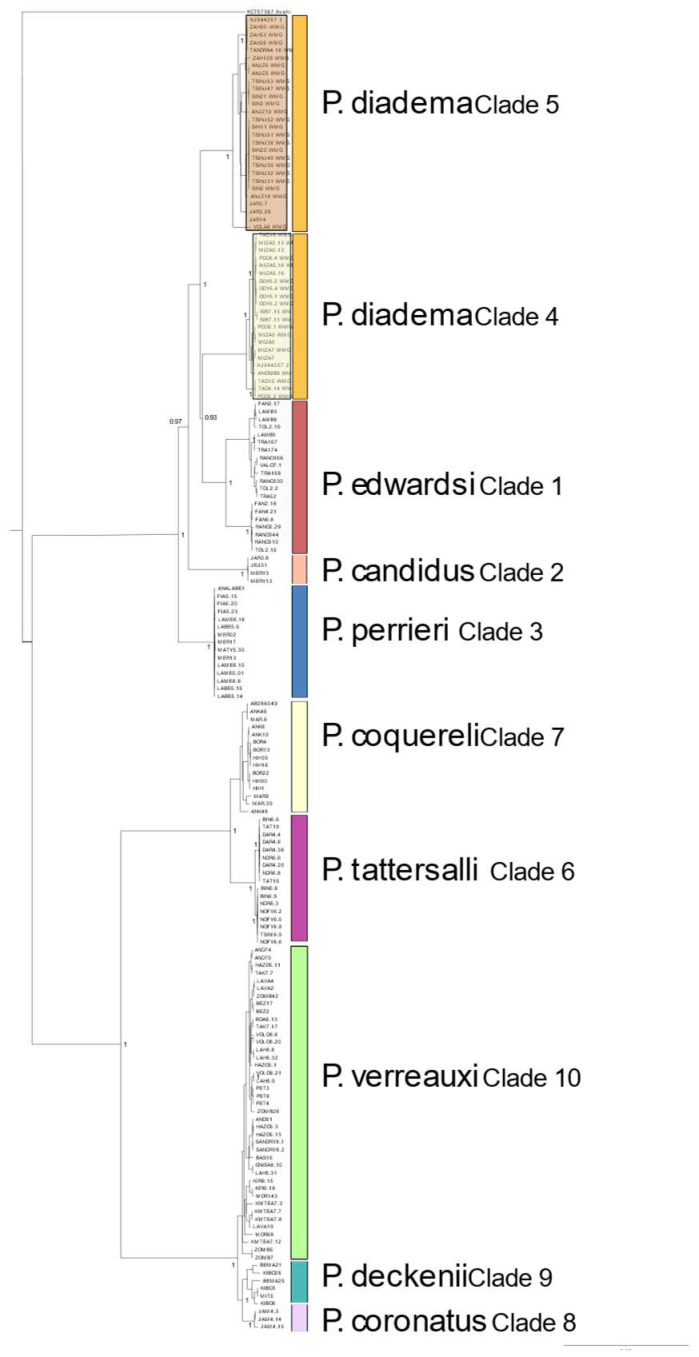
Complete mitochondrial genome phylogenetic tree showing mitochondrial introgression between *P. edwardsi* (clade 1) and *P. diadema* clade 4. All other species were recovered as reciprocally monophyletic. Posterior probabilities shown at nodes.

The recovery of mitogenomes varied across individuals, since mitochondrial sequences were mined from a bycatch of UCE enrichments. A total of 169 new mitogenomes spanning all species of sifakas were generated and combined with three published sequences (GenBank Accession #’s KC757387, AB286049 and KJ944257) resulting in an alignment of 172 individuals which was 16,315 bp in length (Figure 3). Only mitogenomes which had 5X average coverage were included in analyses; details of all samples can be found in Supplementary Table S6. The mitochondrial genome data supported previous phylogenies of the genus [3,4]. The split between the eastern and western species was deep and well-supported, and *P. tattersalli* (clade 6) was placed within the western clade, sister to *P. coquereli* (clade 7). Within *P. verreauxi* (clade 10), three well-supported groups were recovered, with *P. deckenii* (clade 9) and *P. coronatus* (clade 8) sister to *P. verreauxi.* There was no recovered admixture between *P. deckenii* and *P. coronatus* in mitochondria. Within the eastern sifakas, *P. perrieri* (clade 3) was the first to diverge, followed by *P. candidus* (clade 2). *P. diadema* and *P. edwardsi* were admixed, with one group of *P. diadema* branching first (clade 5, composed of the populations from the Tsinjoarivo Forest, the Zahamena National Park, the Anjozorobe–Angavo Protected Area, and the Marotandrano Special Reserve), followed by the sister clades of *P. edwardsi* (clade 1) and *P. diadema* clade 4 (representing the Mantadia National Park, the Mangerivola Special Reserve, the Maromizaha Natural Resource Reserve, the Anosibe An’Ala Forest, and the Sahanody Forest).

The genetic distances across the mitogenome matrix can be found in Supplementary Table S7. Briefly, the recovered CMG divergence between *P. diadema* and *P. verreauxi* was approximately 7.85% divergent, between *P. diadema* and *P. coquereli*: ~7.55%, between *P. diadema* and *P. coronatus/deckenii*: ~7.85%, and between *P. diadema* and *P. tattersalli*: ~7.6%. In the eastern species, the divergence between *P. diadema* and *P. edwardsi*: ~2.25%, between *P. diadema* and *P. candidus*: ~2.4%, between *P. diadema* and *P. perrieri*: ~2.4%, and between the two *P. diadema* clades: ~2.2%.

### 3.5. Extended Bayesian Skyline Plot

EBSP was performed on the two clades of *P. diadema* to evaluate the demographic history of these clades. The coalescent approach was favored over a birth-death process, as the demographic history of these clades was our primary interest. The eight loci EBSP for Clade 4 (which contained the following locations: the Mantadia National Park, the Maromizaha Natural Resource Reserve, the Mangerivola Special Reserve, and the Anosibe An’Ala Forest) showed a fairly sudden population expansion after 20,000 ybp, with an estimated population increasing nearly sevenfold in that time (Supplementary Figure S1a). Clade 5 (containing individuals from the Anjozorobe–Angavo Protected Area, the Tsinjoarivo Forest, the Marotandrano Special Reserve, and the Zahamena National Park) had a more gradual population expansion of about a threefold increase in population size in the same time frame (Supplementary Figure S1b). The four loci dataset (where all four UCE loci were removed) produced an overall similar pattern with a more recent rapid expansion in Clade 4 (Supplementary Figure S1c) and a more gradual expansion in Clade 5 (Supplementary Figure S1d).

### 3.6. Ecological Niche Modeling

**Figure 4:**
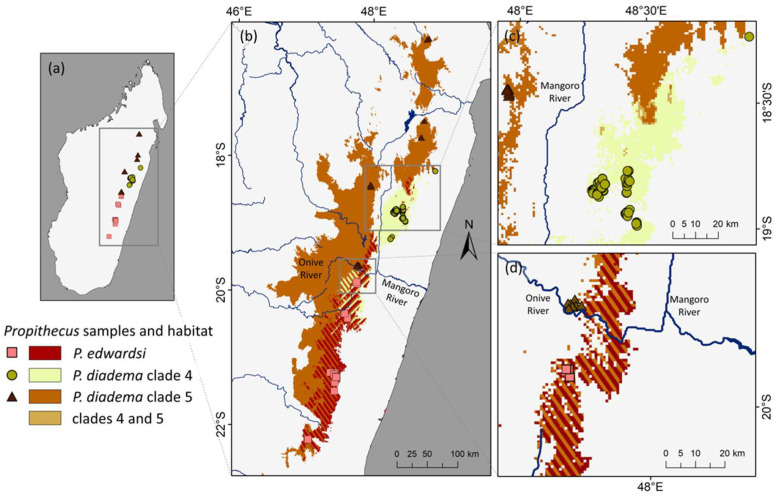
Ecological niche models for the two *Propithecus diadema* clades (4 and 5) as well as *P. edwardsi*. Habitat that is suitable to more than one clade is indicated with hashed lines with the color depicting each clade. (**a**) The samples used in this study. (**b**) The full niche models (not restricted to forest cover) for the three groups across eastern Madagascar. (**c**) Only the forest-limited models for *P. diadema* clades 4 and 5 and indicates a limited potential contact zone of niche overlap, despite the absence of a physical barrier between these taxa. (**d**) Only the forest-limited models for *P. edwardsi* and *P. diadema* clade 5, with suitable habitat for both species found on either side of the Onive River. Recorded occurrences of these taxa indicate that the Onive may act as a dispersal barrier, despite this potentially suitable habitat. The models in (**c**,**d**) are limited to forest cover in 2017, the most recent year available [74].

After recovering two well-supported clades within *P. diadema*, we constructed niche models with high predictive abilities and low levels of overfitting for each clade (Supplementary Table S8; Figure 4). Clade 4 represents all individuals from the following populations, with sample codes given in parentheses: the Mangerivola Special Reserve (VOLA), the Maromizaha Natural Resource Reserve (MIZA), the Mantadia National Park (PDD and TAD), the Anosibe An’Ala Forest (SIB and ANOSIB), and the Sahanody Forest (ODY), shown in purple in all figures. Clade 5 represented individuals from: the Zahamena National Park (ZAH), the Marotandrano Special Reserve (TAND), the Tsinjoarivo Forest (SIN or TSINJ), and the Anjozorobe–Angavo Protected Area (ANJZ), shown in blue in all figures. The ecological niches of both *P. diadema* clades were significantly diverged from each other and from *P. edwardsi* (Identity Test *p* < 0.01; Table 3). In fact, our results show that the ecological niches of *P. diadema* clade 5 and *P. edwardsi* are more similar to each other than to *P. diadema* clade 4. The age overlap correlation test revealed no significant relationship between phylogenetic relatedness and ecological niche similarity, confirming that closely related sifaka taxa do not tend to occupy similar niches (*p* = 0.49).

Precipitation of the driest month (BIO14) was important to both *P. diadema* clades, explaining 62% of variation for clade 5 and 28% for clade 4. However, these lineages differed in their responses: clade 5 occurs in areas with drier dry seasons, and clade 4 occurs in areas with wetter dry seasons (Supplementary Figure S4). These preferences may explain the limited overlap between these clades’ niches in environmental and geographic space. Their potential contact zone (i.e., the forested area northeast of the Mangoro River with suitable habitat for both clades) measures 57.3 km^2^ in total (Figure 4).

## 4. Discussion

### 4.1. Phylogenomics of Sifakas

In this study, we generated the most comprehensive phylogeny of sifakas in terms of both species and geographic distribution of individuals. We recovered largely concordant topologies to previous mitochondrial studies but allow for a much finer-scale interpretation of widespread species. We recovered the same east–west pattern that is well established in the literature and confirmed the placement of *P. tattersalli* with the western sifakas. In most datasets, we established a reciprocal monophyly across nominal taxa. The exceptions are detailed here. In our species tree analyses, we identified that *P. deckenii* and *P. coronatus* appear to hybridize minimally at our sampling locations (Supplementary Figure S3, clades 8 and 9). These findings corroborate field studies of intermediate phenotypes [21,22,86]. While the observed admixture may reflect scenarios of secondary contact following isolation, we cannot discern the degree to which each represents valid and non-interbreeding species. In contrast, the mitochondrial genomes recover reciprocally monophyletic lineages (Figure 3) for *P. deckenii* and *P. coronatus*, as do the concatenated UCEs for the 50 most informative loci (Figure 1).

Unfortunately, our sampling of those two species was poor compared to other species, and, as such, no taxonomic revisions are proposed for this group, as we may have uniquely sampled a hybrid zone. Additional sampling of these two species is warranted to understand the extent of the hybridization, and whether or not classification as separate species is warranted. Morphologically, the skulls of *P. deckenii* and *P. coronatus* skulls are distinct, outside of known areas of hybridization, with the muzzle being shorter and broader in *P. coronatus* when compared with *P. deckenii* [1]. In addition to the admixture identified between *P. deckenii* and *P. coronatus*, we identified mitochondrial introgression between *P. edwardsi* (clade 1) and clade 4 of *P. diadema* (populations including the Sahanody Forest and the Mantadia National Park, among others).

Most other taxa recovered monophyletic groups in both the mitochondrial and nuclear datasets. Mitochondrial introgression was observed between clade 4 of *P. diadema* and *P. edwardsi*, which is explained in further detail below. Multiple well-supported lineages were recovered across the range of *P. verreauxi*, which is unsurprising for a species with such a large distribution. Additional research into the geographic variation of *P. verreauxi* is warranted to fully describe the genetic diversity of this species.

Perhaps most surprising was the identification of cryptic genetic diversity within *P. diadema*. The divergence time between clades 4 and 5 was the same as the divergence between *P. candidus* (clade 2) and *P. perrieri* (clade 3), which were estimated to have diverged 1.7–2.54 million years ago. Clades 4 and 5 also exhibited approximately the same mitochondrial divergence as those species (~2.2% across the entire mitogenome). The two clades of *P. diadema* were also briefly detailed in [24], where they identified the two clades from cytochrome *b* data across most of the same sampling locations as found here. Additional implications of the cryptic divergence within *P. diadema* are described below. Interestingly, no subspecies have been described within this species. A population of melanistic *P. diadema* were identified at Tsinjoarivo, with individuals representing standard coloration to those which appear almost entirely black. However, it has been noted that completely black offspring were found to occur from typically colored parents, implying it is likely to be a single pelage mutation, which is not associated with a divergent subpopulation. From our Tsinjoarivo samples, we did not identify a greater genetic divergence than other populations from clade 5; however, we did recover regional clades containing samples from the northern portion of clade 5 (the Zahamena National Park) to central (the Anjozorobe–Angavo Protected Area and the Marotandrano Special Reserve) and then to the south (the Tsinjoarivo Forest). Despite the known pelage variation at the Tsinjoarivo location, we found no evidence to support previous hypotheses that this location harbors a unique subspecies or one derived from hybrid origin [1].

### 4.2. Species Trees 

The STAR and NJst species trees recovered concordant topologies; however, clades 4 and 5 (*P. diadema*) resulted in some individuals placed into different clades. This could be an artifact of missing data distributed across the hundreds of included UCEs (largely represented by Ns inserted in the more variable and informative flanking regions). It is possible this admixture is a result of the introgression observed via the mitogenomes, and subsequent analyses should be performed to determine if either missing data or introgression/ILS is the causative factor. Other defined species were composed of the same individuals, except for the potential hybrids identified in *P. coronatus* and *P. deckenii* (clades 8 and 9 from Figure 1, Figure 2 and Figure 3) as described above.

### 4.3. Mitochondrial Signatures

The recovered topology was as expected, except in one instance. Interestingly, we recovered a mitochondrial introgression between *P. diadema* clade 4 and *P. edwardsi*. All other nominal taxa were recovered as monophyletic. The mitochondrial introgression within *P. diadema* and *P. edwardsi* was previously described in [24], who attributed the pattern to a male-mediated gene flow followed by secondary contact between the two *P. diadema* clades, leading to nuclear DNA swamping after hybrid clade 4 females reproduced with clade 5 males. This phenomenon explains the observed patterns of a mitochondrial introgression between *P. diadema* clade 4 and *P. edwardsi*, yet less signatures in the nuclear data. Interestingly, Rumpler et al. [24] found both *P. diadema* clades at the Zahamena location (as evidenced from a single specimen, with GenBank Accession # HQ731545), but we did not recover any individuals from the Zahamena National Park with clade 4 haplotypes. Additionally, Rumpler et al. [24] lists two specimens from the same location approximately 17.9 km southeast from the Zahamena samples included here to belong in both clades 4 and 5.

### 4.4. Evolutionary Dynamics

Godfrey et al. [87] found moderate evidence for extinction events and subsequent diversification in Madagascar at 33 Ma, 17 Ma, and 8–9 Ma. This finding has important implications for our study, as we may have support for the 8–9 Ma intrageneric diversification across the E–W clades of sifakas [87]. The 17 Ma occurrence may be associated with the colonization of Madagascar by the euplerid carnivores, which may be linked to the diversification events around that time, potentially including between the *Propithecus* and the *Avahi* [87].

Based on the 10kTree v3 [66] primate tree, there appears to be similar ages of diversification across extant lemurs, with many diversification events occurring at the generic level between 20–25 Ma and intrageneric level between 5–8 Ma. Although quite a large time span for both, the generation times, lifespans, and ecologies of lemurs are vast, and the pattern may correlate with the findings of Godfrey et al. [87]. Additionally, other studies of lemurs have found divergence dates within genera to approximately 15 MYA in *Lepilemur* [88] based only on mitochondrial genomes, as well as around 9–10 MYA for *Eulemur*, *Hapalemur* and *Microcebus* [67].

Our divergence-dating estimates incorporated three different age constraints for the *Avahi–Propithecus* split, with the oldest estimate from [67] at approximately 20 MYA and the youngest from [68] at 13.4 MYA. Due to these various estimates, the divergence dates across the trees varied. The datasets incorporated for each dataset included minimally mitochondrial DNA, with the 10kTrees phylogeny incorporating multiple mitochondrial DNA and one nuclear (IRBP) gene [66] and with four nuclear genes (ADORA3, CNR, RAG1 and RAG2) and two mitochondrial (Cytochrome *b* and ND4) in [67]. Kistler et al. [68] incorporated whole mitochondrial genomes. When incorporating the oldest divergence estimate, the split between the eastern and western taxa coincides nicely to the subtle diversification event detailed in [87].

Despite the variation of ages used for calibration points, the splits between species are all of considerable time-depth for interspecific divergence in primates, including lemurs [89,90,91,92,93,94,95,96].

Even within the two clades of *P. diadema*, the divergence was estimated at minimally 1.74 MYA, a much deeper age estimate than a newly described mouse lemur [97]. While a taxonomic revision of *P. diadema* is beyond the scope of this study, we provide evidence worthy of further morphological evaluation to determine if these cryptic lineages warrant elevation to the subspecific or specific level. The distribution of the two clades is also interesting and provides robust evidence for environmental selection along the climate-zones in eastern Madagascar.

### 4.5. Biogeographic Patterns in P. diadema

The two clades of *P. diadema* exhibit significant ecological divergence from each other and from nearby *P. edwardsi*. Differing tolerances for the precipitation levels of the driest months may contribute to the lack of geographic overlap between the two *P. diadema* lineages, with clade 5 occurring in areas with drier dry seasons and clade 4 in areas with wetter dry seasons. These ecological differences mean that the possible area of contact between these clades is narrow (<60 km^2^) and suggest that current climatic conditions may help maintain genetic isolation between these clades.

However, Rumpler et al. [24] detected both mitochondrial clades in their site in Zahamena, whereas we only found clade 5 in our samples from a nearby location in Zahamena. The species tree reconstructions show some admixture between the clades 4 and 5; however, that may be due to missing data in the UCE dataset. It is also possible that incomplete lineage sorting/introgression has led to these patterns. Additional fine-scale genetic sampling in locations between the two clades may provide further support for the separate clades or identify introgression. Additionally, a comprehensive morphological evaluation should be performed on *P. diadema* across its range to determine if the deep genomic signatures presented here represent divergence worthy of separating this taxon into two distinct yet cryptic species. An evaluation including the holotype of the species is required to test this hypothesis.

Mitochondrial introgression was observed between clade 4 of *P. diadema* and *P. edwardsi*, and *P. edwardsi* and *P. diadema* clade 5 exhibit a geographic overlap between their ecological niches. However, the current distributions of *P. edwardsi* and *P. diadema* clade 5 appear to be delimited by the Onive River (Figure 4). The Onive may act as a modern barrier to dispersal for these taxa. Multiple mechanisms for maintaining reproductive isolation (e.g., ecological divergence, allopatric barriers) could be at work in the eastern sifakas. Presently we do not know the historical climate and riverine locations of these lineages and, as such, understanding of the ancestral distributions is unknown.

## 5. Conclusions

In this paper, we have provided the first nuclear, and by far the most comprehensive, phylogeny of sifakas. We have dated the splits between species based on a large nuclear dataset and confirmed the early divergence between the eastern and western groups. We have also confirmed the ancient split of *P. tattersalli* from other western species around 4.7–6.2 MYA, followed by *P. coquereli*, then *P. verreauxi*, and finally the questionable species-level designation of *P. deckenii* and *P. coronatus*. Further sampling across the range of these sifakas is critical to understanding whether the pelage and morphological variation in these taxa is sufficient to classify them as separate species. The eastern sifakas recovered an early split between *P. edwardsi* and all other species, followed by the splitting of *P. diadema* from *P. candidus* and *P. perrieri*. *P. diadema* recovered two well-differentiated clades with divergent ecological niches and no modern geographic overlap. Clade 4 of *P. diadema* had a closer mitochondrial relationship with *P. edwardsi* than the other clade (5) of *P. diadema*, a phenomenon potentially resulting from mitochondrial introgression from male-biased dispersal. The two clades of *P. diadema* have not been described as subspecies in the past, although we cannot state for certain whether or not *P. diadema* contains two cryptic taxa without additional morphological analyses.

Regardless of the taxonomy, the conservation status of all sifakas is dire. All nominal species are now considered Critically Endangered, and here we date the evolutionary divergence between them over 1.7 MYA (based on the youngest reconstructions and including the intraspecfic split found in *P. diadema*). The current rate of deforestation in Madagascar is unsustainable, and these unique lemurs are decreasing across the island. Additionally, very few sifakas exist in captive collections as husbandry has been challenging, so no substantial rescue populations are available for any species. *P. perrieri* is projected to have fewer than 50–230 individuals remaining across all populations [98]. As such, protecting the remaining habitat and promoting connectivity seems the best way to allow for their survival in perpetuity. Beyond lemurs, Madagascar is known for its incredibly high rates of endemism, and, if other taxa have similar patterns of divergence across these climate zones, it may be a race against time to characterize these patterns and protect the remaining forest fragments.

## Figures and Tables

**Table 1:** Summary of the number of individuals across each species and locations included in this study. Additional details can be found in Supplementary Table S1.

n	Species	Specific Locations
8	*P. candidus*	Anjanaharibe-Sud Special Reserve, Marojejy National Park
15	*P. coquereli*	Anjiamangirana Forest, Ankarafantsika National Park, Bora Forest, Mariarano Forest
6	*P. coronatus*	Anjahamena Forest
8	*P. deckenii*	Tsingy de Bemaraha National Park, Tsiombikibo Forest
48	*P. diadema*	Mantadia National Park, Anjozorobe-Angavo Protected Area, Anosibe An’Ala Forest, Mangerivola Special Reserve, Maromizaha Natural Resource Reserve, Marotandrano Special Reserve, Sahanody Forest, Tsinjoarivo Forest, Zahamena National Park
25	*P. edwardsi*	Andringitra National Park, Fandriana Forest, Marolambo National Park, Ranomafana National Park, Tolongoina Forest
17	*P. perrieri*	Analamerana Special Reserve, Andrafiamena Andavakoera Protected Area
22	*P. tattersalli*	Andranotsimaty Forest, Antobinitsimihety Forest, Binara Forest, Bobankora Forest, Daraina (Matamena Forest), Mahabenofy Forest
59	*P. verreauxi*	Analalava Forest, Andohahela National Park, Ankaboa Forest, Ankilelignisa Forest, Menabe Antimena Protected Area, Bezà-Mahafaly Special Reserve, Ifotaka Forest, Isalo National Park, Kirindy Mité National Park, Lavavolo Forest, Manakaralahy Forest, Matsandry Atsimo Forest, Tsimanampetsotsa National Park, Zombitse-Vohibasia National Park
	**outgroups**	
2	*Lemur catta*	Anakaboa Forest
1	*Indri indri*	Zahamena National Park
1	*Avahi peyrierasi*	Ranomafana National Park
Total:	212	Including outgroups

**Table 2:** Divergence date comparisons with three different age constraints for the *Avahi–Propithecus* split [66,67,68]. Node age is shown with 95% HPD in parentheses, and all ages are shown in millions of years.

	10KTrees [66]	Herrera & Davalos [67]	Kistler et al. [68]
*Avahi-Propithecus* calibration	16.5	19.99	13.4
recovered date:	16.38 (14.38–18.32)	19.82 (17.86–21.79)	13.25 (11.24–15.17)
East-West divergence	7.6 (5.71–9.53)	8.99 (7.14–11.06)	6.46 (4.53–7.58)
Western Sifakas			
*P. tattersalli*	5.27 (3.92–6.59)	6.24 (4.84–7.64)	4.71 (3.03–5.46)
*P. coquereli*	4.12 (2.97–5.12)	4.76 (3.71–5.99)	3.5 (2.12–4.97)
*P. verreauxi*	3.14 (2.23–3.9)	3.62 (2.76–4.64)	2.68 (1.49–3.64)
*P. coronatus/P. deckenii*	2.26 (1.55–2.86)	2.61 (1.88–3.4)	1.89 (1.11–2.61)
Eastern Sifakas			
*P. edwardsi*	3.72 (2.77–4.74)	4.44 (3.78–5.54)	3.31 (1.88–5.36)
*P. diadema- P. candidus/P. perrieri*	3.06 (2.27–3.91)	3.68 (2.87–4.59)	2.61 (1.64–3.54)
*P. candidus- P. perrieri*	2.11 (1.49–2.77)	2.54 (1.86–3.3)	1.7 (1.15–2.38)

**Table 3:** Two metrics comparing the overlap between ecological niches for *P. diadema* and *P. edwardsi*. Schoener’s D is represented in the upper right triangle of the table and Hellinger’s I in the lower left. Both metrics range from 0 (no overlap) to 1 (identical). Pairs that were shown to be significantly different using the Identity Test are marked with an asterisk [84].

	*P. diadema clade 5*	*P. diadema clade 4*	*P. edwardsi*
*P. diadema clade 5*	1	0.23 *	0.10 *
*P. diadema clade 4*	0.49 *	1	0.24 *
*P. edwardsi*	0.26 *	0.51 *	1

## Data Availability

DNA sequence alignments, tree files, and input and configuration files can be found at the listed FigShare link: https://smithsonian.figshare.com/projects/Phylogenomics_of_the_sifakas/96905 accessed on 1 January 2020. Raw sequence data has been uploaded to GenBank’s Short Read Archive under the following title: BioProject PRJNA803363 and BioSamples Accession #’s: SAMN25649035- SAMN25649239. Complete mitochondrial genomes have been deposited on GenBank under the following Accession numberss: OM649948- OM650112. Nuclear genes associated with the EBSP analysis are available at the following Accession numbers: ON023122-ON023122.

## Supplementary Materials

The following supporting information can be downloaded at: https://www.mdpi.com/article/10.3390/genes13061026/s1. Supplementary Table S1: List of all individuals included in this study and GPS of each general location. Supplementary Table S2: Complete barcode combinations used for this project using Illumina iTru style adapters as ordered from Glenn (2016). Supplementary Table S3: Average Complete Mitogenome coverage across all novel mitogenomes generated here. The average, minimum, and maximum coverage, as well as the number of reads mapped are shown. Only individuals with an average coverage of 5× or greater were included. Supplementary Table S4: Partitions recovered from the SWSC-entropy models for the 50 most informative loci. Supplementary Table S5: Model parameters and evaluation metrics. AUC values range from 0–1, with AUC = 0.5 indicating no predictive ability and higher AUC values indicating better predictive accuracy. Supplementary Table S6: Read counts of PE reads from UCE enrichments. Supplementary Table S7: CMG Distances Number of recovered reads per individual following UCE enrichment. Note: samples with less than 40,000 PE reads (highlighted in red) were excluded from analysis with the phyluce pipeline. Supplementary Table S8: Model parameters and evaluation metrics. Supplementary Figure S1: Extended Bayesian Skyline Plot. Supplementary Figure S2: ‘Get informative sites’ results plotted from all enriched UCEs. The script is available as part of the phyluce pipeline [49]. Supplementary Figure S3: NJst species tree reconstruction from 512 UCE loci. Note the topology shows admixture between the *P. coronatus* and *P. deckenii* (clades 8 and 9 in Figure 1, Figure 2 and Figure 3) where teal individual names represent *P. deckenii* and black text represent *P. coronatus*, as well as between the two *P. diadema* clades (#’s 4 and 5). Individuals are colored by the respective clades (light and dark orange) as completed in all figures. The apparent admixture between in *P. diadema* may be due to missing data in the sequence matrix. Supplementary Figure S4: Marginal response curves for *P. diadema* clades 4 and 5 and *P. edwardsi* to each variable used in constructing their ecological niche models. The percentages indicate that variable’s contribution to the overall niche model.
