# Supplementary material for: Nuclear and Mitochondrial Phylogenomics of the Sifakas Reveal Cryptic Variation in the Diademed Sifaka
Source: Genes (Basel). 2022 Jun 7;13(6):1026. doi: 10.3390/genes13061026 (PMC9223071; doi:10.3390/genes13061026)
Supplement: Supplementary file 1 [file genes-13-01026-s001.zip › figure.pdf]

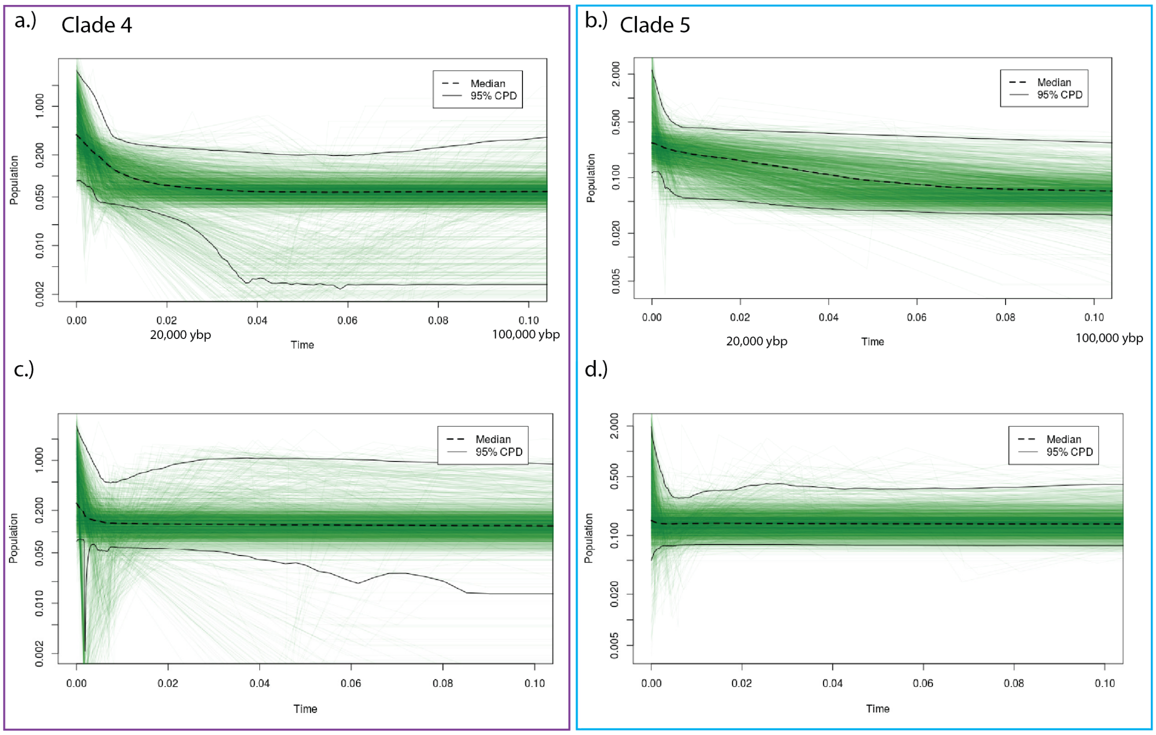

Supplementary Figure S1: Extended Bayesian Skyline Plot.

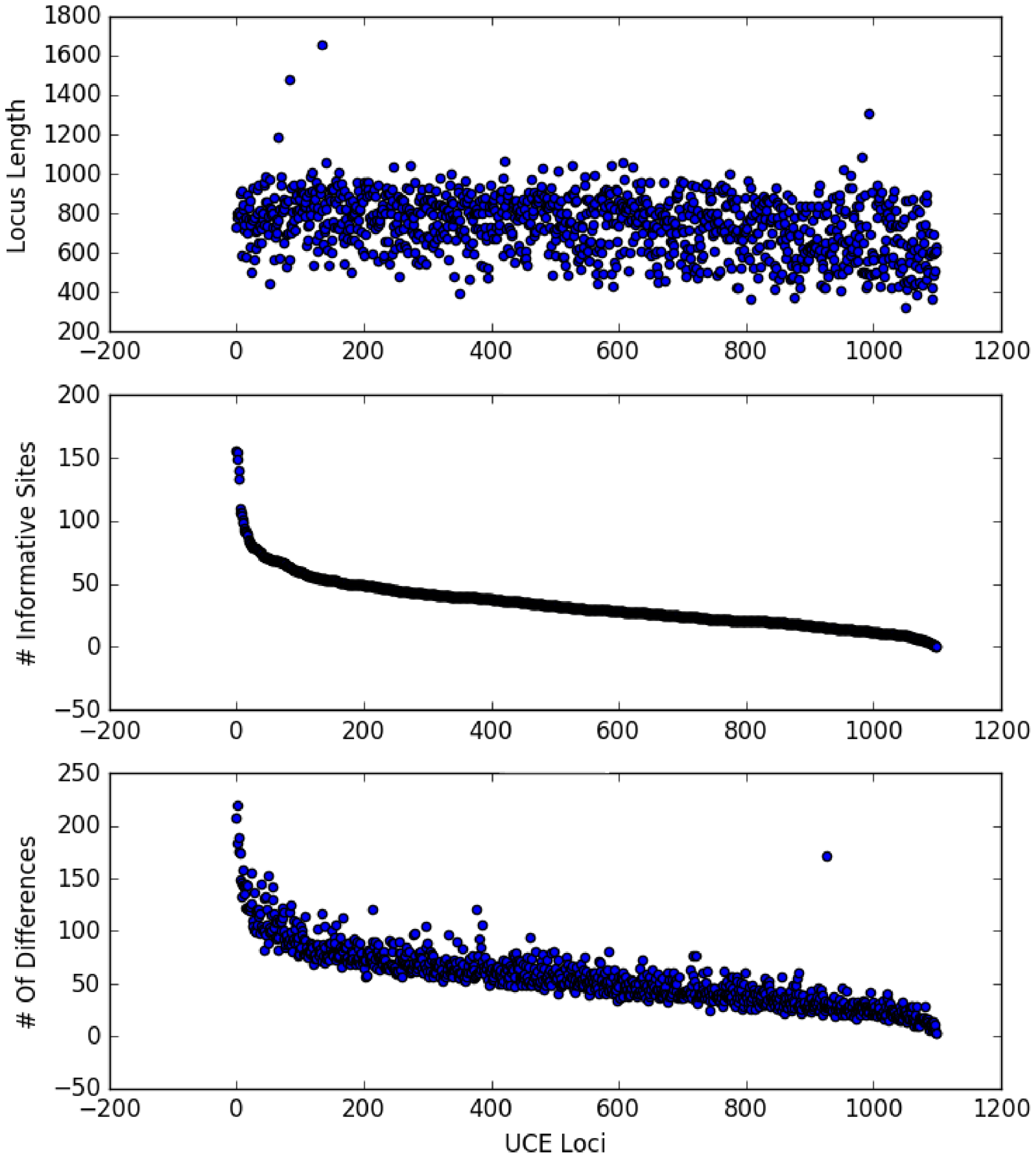

Supplementary Figure S2: 'Get informative sites' results plotted from all enriched UCEs. The script is available as part of the phyluce pipeline [49].

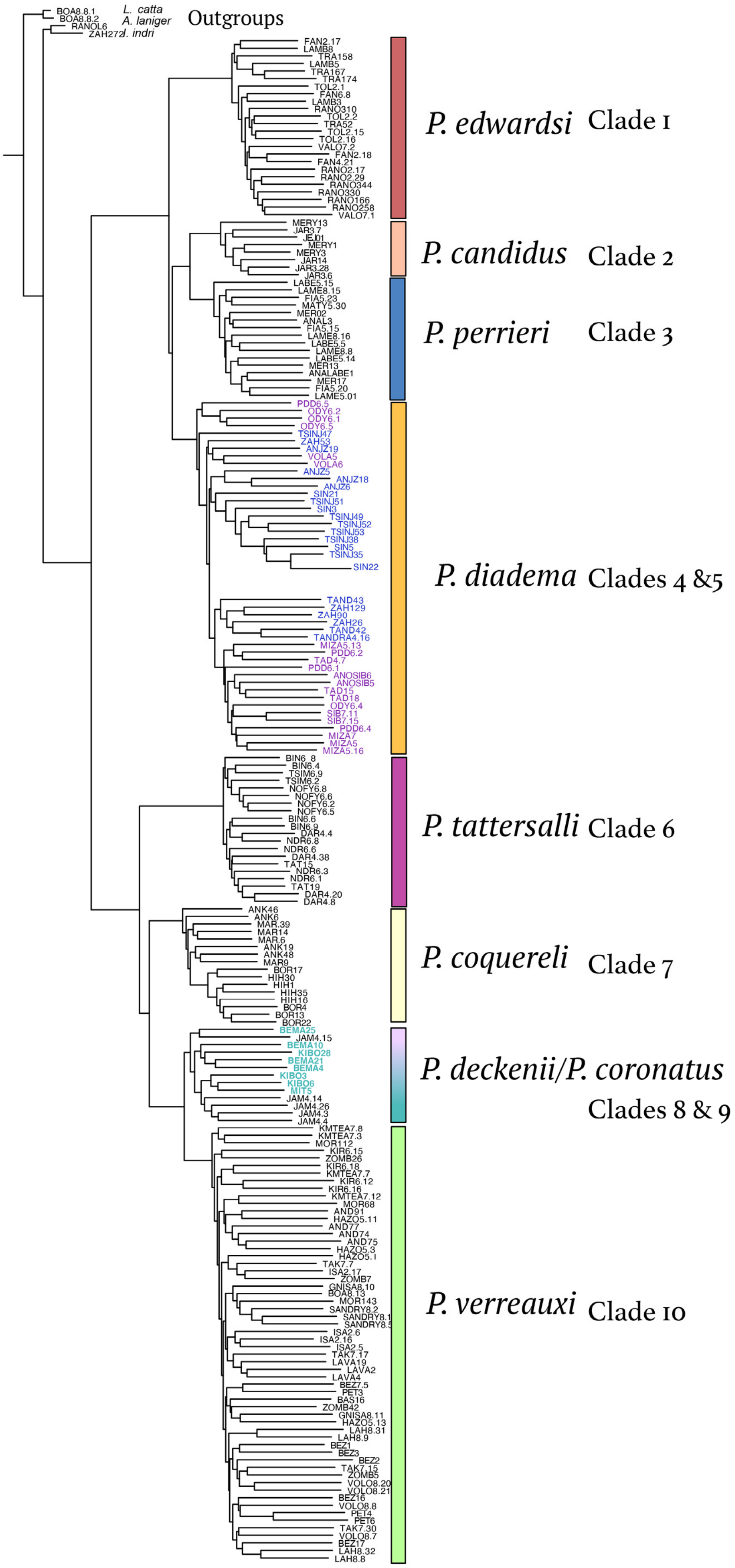
L. catta
A. laniger
I. indri
Outgroups
P. edwardsi
Clade 1
P. candidus
Clade 2
P. perrieri
Clade 3
P. diadema
Clades 4 &5
P. tattersalli
Clade 6
P. coquereli
Clade 7
P. deckenii/P. coronatus
Clades 8 & 9
P. verreauxi
Clade 10

Supplementary Figure S3: NJst species tree reconstruction from 512 UCE loci. Note the topology shows admixture between the *P. coronatus* and *P. deckenii* (clades 8 and 9 in Figures 1–3) where teal individual names represent *P. deckeni*i and black text represent *P. coronatus*, as well as between the two *P. diadema* clades (#'s 4 and 5). Individuals are colored by the respective clades (light and dark orange) as completed in all figures. The apparent admixture between in *P. diadema* may be due to missing data in the sequence matrix.

Propithecus diadema clade 4

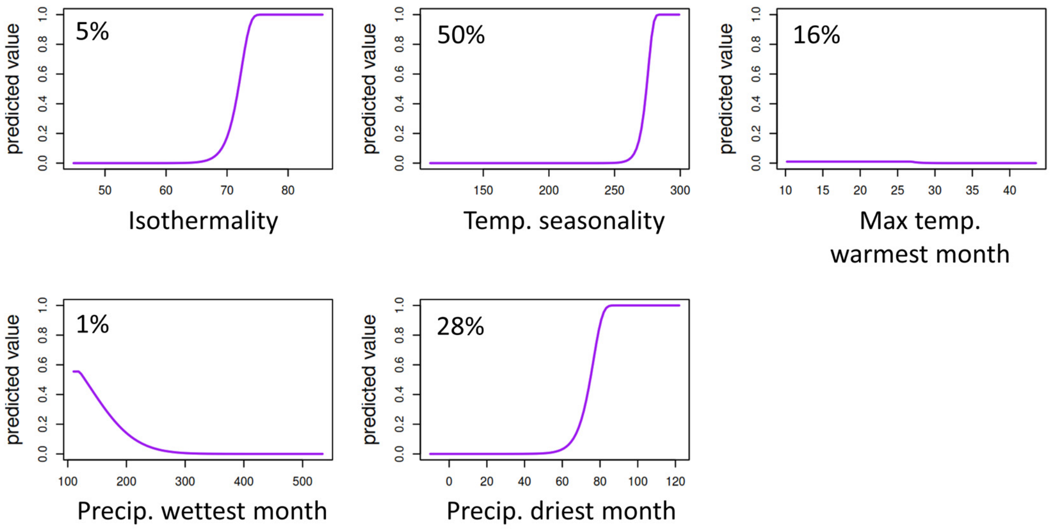

Propithecus diadema clade 5

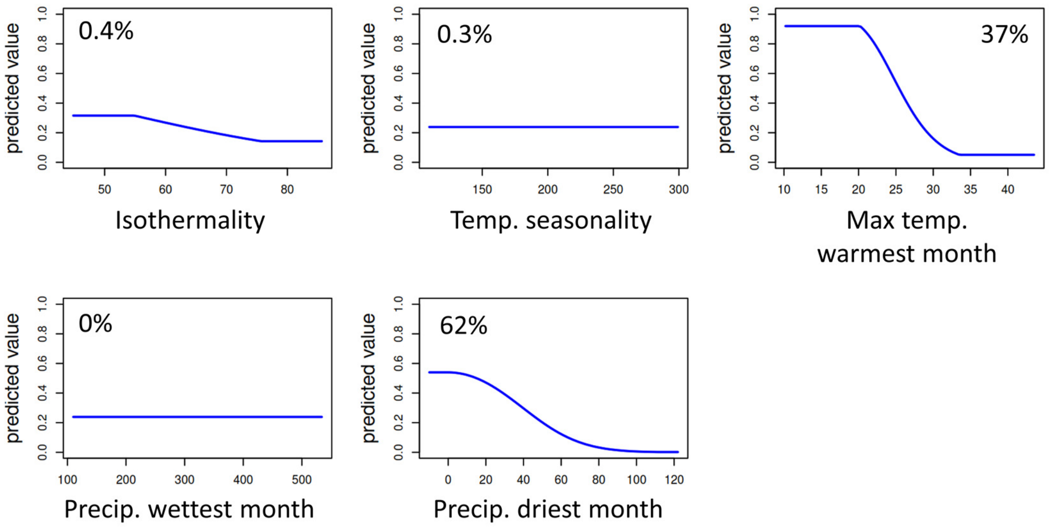

Propithecus edwardsi

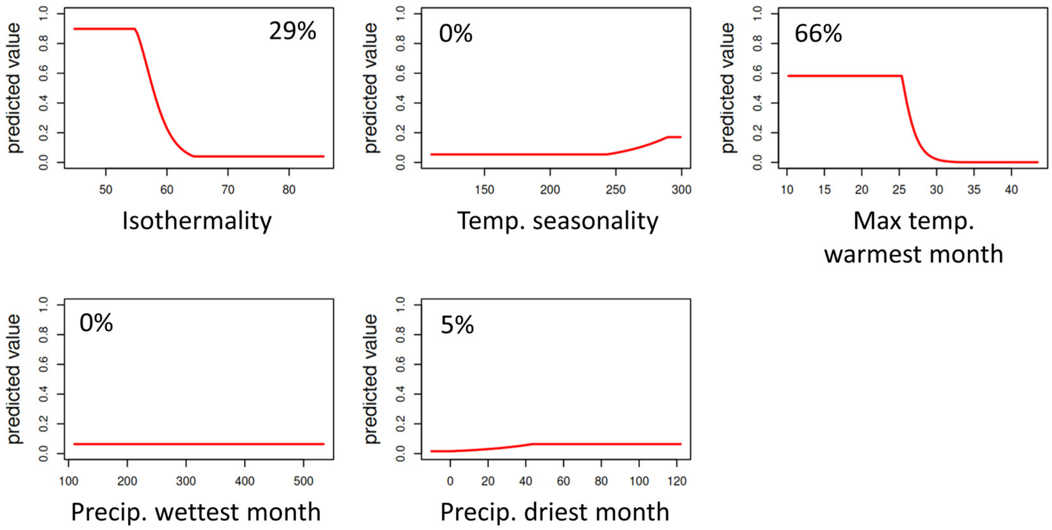

Supplementary Figure S4: Marginal response curves for *P. diadema* clades 4 and 5 and *P. edwardsi* to each variable used in constructing their ecological niche models. The percentages indicate that variable's contribution to the overall niche model.
